# Spatiotemporal changes in exposition risk to leishmaniases vector in residences within a fishing tourism area of Pantanal wetland

**DOI:** 10.1371/journal.pntd.0011809

**Published:** 2023-12-04

**Authors:** Daiana Alovisi Souza, Luiz Gustavo Rodrigues Oliveira-Santos, Jucelei de Oliveira Moura Infran, Wagner de Souza Fernandes, Aline Etelvina Casaril Arrua, Eliane Mattos Piranda, Alessandra Gutierrez de Oliveira

**Affiliations:** 1 Graduate Program in Animal Biology, Institute of Biosciences, Federal University of Mato Grosso do Sul, Campo Grande, Brazil; 2 Graduate Program in Ecology, Institute of Biosciences, Federal University of Mato Grosso do Sul, Campo Grande, Brazil; 3 Laboratory of Human Parasitology, Institute of Biosciences, Federal University of Mato Grosso do Sul, Campo Grande, Brazil; 4 Graduate Program in Infectious and Parasitic Diseases, Faculty of Medicine, Federal University of Mato Grosso do Sul, Campo Grande, Brazil; University of California Davis School of Veterinary Medicine, UNITED STATES

## Abstract

Miranda Municipality of Mato Grosso do Sul, borders the Pantanal wetland, a famous fishing destination visited by tourists from all over the world, and is a location where visceral leishmaniasis has been reported. To assess the risk of *Leishmania infantum* transmission, we studied the sandfly community, focusing on known vector and parasite presence. We conducted light trap collections twice per month at nine sites within the city (including two forested areas) for one year. We collected a total of 12,727 sand flies, 10,891 males and 1,836 females belonging to 11 species: *Brumptomyia avellari*, *Evandromyia aldafalcaoae*, *Ev*. *evandroi*, *Ev*. *lenti*, *Ev*. *sallesi*, *Ev*. *walkeri*, *Lu*. *longipalpis*, *Nyssomyia whitmani*, *Psathyromyia bigeniculata*, *Pa*. *hermanlenti* and *Pa*. *punctigeniculata*. *Lutzomyia longipalpis*, the proven vector of *Leishmania infantum*, was captured each month, and was the most abundant species observed, accounting for more than 99% of sand flies captured in most sites, especially where chicken coops were present. Evidence of *Leishmania infantum* infection was detected in 0.40% of *Lu*. *longipalpis* tested. We developed a generalized mixed multilevel model for *Lu*. *longipalpis*, that includes within-year seasonality, location of capture (indoors vs. outdoors), vector abundance, and sex ratio. The VL vector was abundant both inside and outside houses. Large numbers of *Lu*. *longipalpis* were observed in outdoor sites where domestic animals were present but were absent from forest sites. Our findings suggest high vector populations and *Le*. *infantum* presence in a city where tourists could be exposed to visceral leishmaniasis, with significant implications for more surveillance and control activities.

## Author summary

Several localities in Mato Grosso do Sul have already recorded human visceral leishmaniasis cases, including the city of Miranda, which is a famous fishing and tourism destination. Due to the high number of visitors, we decided to evaluate the risk of *Leishmania infantum* transmission in the area. Thus, we conducted vector collections over one year, detecting both the presence of the main vector (*Lutzomyia longipalpis*) and the *Le*. *infantum* parasite. We used nonlinear multilevel mixed models, under the Bayesian framework, to investigate the seasonal changes on transmission risk (i.e.; overall vector abundance and the proportion of females), taking into account the residence space. We found that the overall vector abundance outdoors was higher than in the indoors. Outdoors abundance was more consistent with two-peaks during the year, whereas indoors abundance was more seasonal with a single peak. Although, indoor abundance was lower than observed outdoors, the proportion of females captured was up to twice that observed outdoors. These findings highlighted the spatiotemporal component of leishmaniasis transmission risk, which can guide entomological surveillance and control measures.

## Introduction

Leishmaniases are cosmopolitan anthropozoonoses where protozoan pathogens from the genus *Leishmania* are transmitted between hosts by sand fly vectors belonging to the Phlebotominae subfamily [1,2]. The most common vectors of *Leishmania* (*Leishmania*) *infantum*, the etiologic agent of Visceral Leishmaniasis (VL), are *Lutzomyia longipalpis* and *Lutzomyia cruzi*, are present in state of Mato Grosso do Sul (MS), Brazil [3,4]. The former is distributed throughout Latin America, while the latter is mainly concentrated in central-western Brazil, but it was also recorded in the state of Ceará. In urbanized areas, both species exhibit anthropophilic and endophilic behavior [5–9].

*Lutzomyia longipalpis* is observed year-round, but its abundance increases mainly after rainy periods [10]. Therefore, vector control is necessary year-round due to the risk of transmission. In MS, this disease is endemic, with 1,605 reported cases and 112 deaths between 2010 and 2017. Miranda municipality is located in Pantanal Sul- Matogrossense, an area famous for fishing tourism with the sporadic transmission of VL, with 14 reported cases between 2010 and 2017 [11]. This area has a high human population density, mostly due to a growing tourism industry with many Brazilians (from all regions of the country) and international visitors. The epidemiology of VL and its Public Health burden is not well understood in this unique location, where tourism may play a role in disease transmission [9].

Knowing if the vector is present, and at what density in the locality is mandatory for health surveillance. Our study’s goal was to conduct a risk assessment for VL in Miranda community by 1) characterizing sand fly species diversity and abundance, especially in urban sites, 2) confirming the presence of the VL vector (*Lutzomyia longipalpis*), and the etiologic agent (*Leishmania infantum*), 3) describing VL vector dominance, and how its abundance correlates with other sandflies’ abundance, and 4) characterize within-year seasonal patterns of VL vectors in the area both inside and outside residences.

## Methods

### Study area

The municipality of Miranda (20° 14’ 26” S, 56° 22’ 42” W) located in Pantanal Sul-Matogrossense region, West-central of Brazil [12], belongs to the Paraguay watershed and Miranda sub-watershed. It includes rivers and flooded plains, forest, and woodland savanna [13]. The climate is tropical subhumid, with a relative humidity of 82% and annual average temperature ranging between 22°C and 27°C and rainfall between 800 and 1,200mm [14].

We selected sites to carry out sand fly collections in seven neighborhoods and two peri urban areas based on the locations of reported VL cases, and the presence of domestic (dogs, chicken, goats, cattle, cats, geese, and ducks) or sylvatic reservoirs of *Leishmania* spp. (S1 File).

### Sand fly collection and processing

From August 2013 to July 2014, we carried out sand fly collections using two Falcon-type automatic light traps [15] at each of the 9 sites, twice per month, from 6 p.m. to 6 a.m. At each site one trap each was placed indoors and outdoors. Captured insects were transported to the Laboratory of Parasitology at the Federal University of Mato Grosso do Sul for further processing. Males were processed according to the method proposed by Forattini [16]. We quickly identified the females, allowing the insect to be stored -20C for further molecular analysis. Species identification was performed based on structures of the head, thorax, and abdomen using the taxonomic key of Galati [17] and abbreviations of the genera of sandflies according to Marcondes [18].

Meteorological data were obtained by the Center of Weather, Climate, and Water Resources monitoring [19]. Sampling was carried out under the permanent license for the collection of zoological material issued by the Brazilian Institute of the Environment and Renewable Natural Resources (IBAMA: SISBio 25592–2).

### Detection of *Leishmania* spp

Non-engorged females were placed individually, or in pools of up to 10 individuals, for later testing by Polymerase Chain Reaction (PCR) to identify *Leishmania*. The DNA was extracted using 5% resin, a copolymer of styrene and divinylbenzene that contains paired iminodiacetate ions according to Loxdale and Lushai [20].

Polymerase chain reaction was performed aiming at the internal transcribed spacer (ITS) region of ribosomal DNA with approximately 300 base pairs (bp) of *Leishmania* sp. It was added 5 μL of a sample, 12.5 μL of a premixed ready-to-use solution containing bacterially derived Taq DNA polymerase, dNTPs, MgCl2, and reaction buffers, 5.5 μL of water, and 1 μL of each oligonucleotide: LITSR (5’-CTGGATCATTTTCCGATG-3’) e L5.8S (5’-TGATACCACTTATCGCACTT-3’) to the final volume of 25 μL [21].

The amplification conditions were 95°C per 3 minutes, followed by 34 cycles of 95°C per 30 seconds, 53°C per 30 seconds, 72°C per 1 minute, with post-extension of 72°C per 5 minutes, in a thermocycler BIOER XP Cycler. It used water for negative control and *Leishmania infantum DNA* (MHOM/BR/1972/BH46) was for positive control [22].

The amplicons were analyzed through agarose gel electrophoresis 1.5% in TBE buffer and Nucleic Acid Stain (10,000X DMSO). The positive samples were analyzed by restriction fragment length polymorphisms (RFLP) with HAE III. The restriction profile was analyzed by agarose gel 2% and compared with the pattern obtained for *Leishmania amazonensis* and *Le*. *infantum* [23]. The minimum rate of infection of sand flies was calculated according to the following formula: minimum rate (MIR) = ([number of positive pools /total number of specimens tested] x100) [24].

### VL vector dominance and correlation with other sandfly species

We investigated how other sandfly species’ abundance (OSF) correlates with VL vector abundance using a Generalized Negative Exponential Mixed Multilevel Model, controlling for natural temporal abundance changes in OSF throughout the year, under a Bayesian framework [25]. Therefore, we regressed the abundance of other sandfly species (OSF) against the VL vector abundance and month of sampling. Because the response variable (OSF abundance) is a counting process of the number of OSF in a certain month (*m*) at a given location (l) sampled in the residence space (*d*), we assumed it follows a Poisson distribution (Eq 1):

$${OSF}_{m,l,d}∼Poisson(\lambda_{m,l,d})$$ Eq 1

where *λ*_*m*,*l*,*d*_, the OSF counting observed in a month *m* at a given location *l* sampled in the domicile space *d*, can be modeled as a negative exponential equation as follows (Eq 2):

$$\lambda_{m,l,d}=\beta_{1}+e^{\beta_{2}*VL+\beta_{3}*month+\beta_{4}*month^{2}}$$ Eq 2

where *β*_1_ depicts the overall mean of OSF abundance, *β*_2_ the effect of VL vector abundance, and *β*_3_ e *β*_4_ the linear and quadratic temporal effect of month. Still, we were also interested in the variation of OSF abundance across locations, then we modified Eq 3, and considered that locations could present different OSF abundance (*β*_1_) by including the location identity as random intercept (“mixed effect”):

$$\lambda_{m,l,d}={(\beta }_{1}+\beta_{l})+e^{\beta_{2}VL+\beta_{3}month+\beta_{4}month^{2}}$$ Eq 3

$$\beta_{l}∼Normal(\beta_{1},\sigma_{\beta_{1}}),$$ Eq 4

where *β*_1_ will depicts the grand population intercept (overall OSF abundance), and *β*_*l*_ is a unique OSF abundance estimated for each location. Note that OSF estimated for each location are interdependent because they come from a variance component $\left(\sigma_{\beta_{1}}\right)$ that was estimated centered on the grant population intercept (*β*_1_) (Eq 4).

Finally, we applied a multilevel approach to checking for effects of residence space (i.e.; outdoor VS indoor) on OSF abundance (*β*_1_) at each location *l*:

$$\beta_{l}=\beta_{1}+\beta_{6}{residence}_{l},$$ Eq 5

where *β*_6_ indicates the estimated difference of OSF abundance between outdoor and indoor.

### Spatio-temporal variation of VL exposition risk

We measured risk of exposure through two metrics: total VL vector abundance and proportion of females. We believe that these metrics can be understood as proxies of risk, and then we modeled how they varied across the residence space (indoor VS outdoor) throughout the year. We followed the analytical mainstream described above (generalized mixed multilevel model), but we used a non-linear circular equation rather than an exponential form because we are dealing with seasonal variability of VL vector abundance and the proportion of females.

We departed from a nonlinear trigonometric equation to model seasonality within a year [25]:

$$f(x)=K+Ecos(\frac{xR\pi }{12})$$ Eq 6

Eq 6 is very convenient for investigating seasonal patterns by describing a wave-like shape using the cosine function. The parameters K, E, and R depict the intercept, amplitude, and frequency of this wave, which in turn can be easily translated into biological terms. For example, if *x* represents a time unit (e.g.; month) within a year, and *f(x)* the VL vector abundance found in that time unit; K, E, and R would inform the yearly mean abundance, the seasonality strength (difference between peaks and valleys of the wave), and the number of abundance peaks within the year, respectively.

The risk metrics used here (abundance and proportion) come from different distributions. VL vector abundance (counting) can be assumed as a Poisson process (Eq 7) as explained above; on the other hand, the proportion of females, that is the number of females observed in a given number of individuals caught in the month *m* at location *l* sampled at domicile space *d*, can be assumed a Binomial process (Eq 8):

$${VL\;vector\;abundance}_{m,l,d}∼Poisson(\lambda_{m,l,d}),$$ Eq 7

$${Number\;of\;females}_{m,l,d}∼Bin({Number\;caught\;individuals}_{m,l,d},p_{m,l,d}),$$ Eq 8

where *p*_*m*,*l*,*d*_ represents the chance of one individual caught in the month *m*, at location *l*, and residence space *d*, be a female.

Both VL vector abundance (*λ*_*m*,*l*,*d*_) and the probability to find a female (*p*_*m*,*l*,*d*_) can be modeled following the seasonal wave described in Eq 6:

$$\lambda_{m,l,d}=K+Ecos(\frac{{month}_{l,d}R\pi }{12})$$ Eq 9

$$logit(p_{m,l,d})=K+Ecos(\frac{{month}_{l,d}R\pi }{12})$$ Eq 10

As did early, we also incorporated in the Eqs 9 and 10 the location identity as a random intercept to account for the spatial heterogeneity among sampling locations in the overall yearly risk (*K*):

$$\lambda_{m,l,d}=(K_{0}+K_{l})+Ecos(\frac{{month}_{l,d}R\pi }{12})$$ Eq 11

$$logit(p_{m,l,d})=(K_{0}+K_{l})+Ecos(\frac{{month}_{l,d}R\pi }{12})$$ Eq 12

$$K_{l}∼Normal(K_{0},\sigma_{k_{o}}),$$ Eq 13

where *K*_0_ depicts the grand mean population intercept of risk (VL vector abundance or proportion of females) and *K*_*l*_ is a unique risk estimation for each location. The multilevel approach was ultimately applied to test if the local risk (*K*_*l*_) depends on the domicile space *d*:

$$K_{l}=K_{0}+\beta_{1}{residence\;space}_{l},$$ Eq 14

where *β*_1_ indicates the estimated difference of risk between indoors and outdoors.

### Bayesian setting and model solving

We solved the above-mentioned models using the Bayesian approach available in the *brms* package. For each model, we ran three Monte Carlo Markov Chains with 3000 iterations each and burned the first half of iterations of each chain. We checked if chains converged and merged along each estimated parameter. We used noninformative priors for each fixed parameter (K_0_, *E* and β_etas_), except for *R*, that was limited to few, positive number of peaks:

$$K_{o},E,\beta_{etas}∼Normal(10^{5},10^{5}),$$ Eq 15

R∼Normal(1,0.5,minimum=0), Eq 16

## Results

After 4,992 hours of sample effort, we collected 12,727 sandflies belonging to three subtribes, five genera, and 11 species (Table 1). Our trap failure rate was low (5% due to battery failure). Overall, the male/female ratio was 6.0 for *Lu*. *longipalpis*, 0.83 for *Evandromyia aldafalcaoae*, 0.75 for *Ev*. *evandroi* and 0.6 for *Ev*. *sallesi*.

**Table 1:** Sandflies species distribution captured with Falcon-type automatic light trap. By genera, site and residence space, Miranda, Mato Grosso do Sul, Brazil, from August 2013 to July 2014.

Species	Cherogamim				Cohab				Baiazinho				Serraria				Ma. Rosário				Nova Miranda				Centro				B. Rio		Mata		Total		Total	%
	IN		OU		IN		OU		IN		OU		IN		OU		IN		OU		IN		OU		IN		OU		OU		OU					
	♂	♀	♂	♀	♂	♀	♂	♀	♂	♀	♂	♀	♂	♀	♂	♀	♂	♀	♂	♀	♂	♀	♂	♀	♂	♀	♂	♀	♂	♀	♂	♀	♂	♀		
*Br*. *avellari*	−	−	−	−	1	−	−	−	−	−	−	−	−	−	1	−	−	−	−	−	−	−	−	−	−	−	−	−	−	−	−	−	2	−	2	0.015
*Ev*. *aldafalcaoae*	−	−	−	−	1	−	1	1	−	−	−	−	−	1	−	−	1	3	1	1	1	−	−	−	−	−	−	−	−	−	−	−	5	6	11	0.086
*Ev*. *evandroi*	−	−	−	−	2	−	−	1	−	−	−	−	−	2	−	−	−	−	1	−	−	1	−	−	−	−	−	−	−	−	−	−	3	4	7	0.055
*Ev*. *lenti*	−	−	−	−	4	−	6	−	−	−	−	1	−	−	−	1	−	−	−	−	−	1	−	2	−	−	−	−	−	−	−	−	10	5	15	0.118
*Ev*. *sallesi*	−	−	1	−	−	−	−	−	−	−	1	−	−	−	−	2	−	−	−	−	−	−	−	−	−	−	−	−	−	1	−	−	2	3	5	0.039
*Ev*. *walkeri*	−	−	−	−	−	−	−	−	−	−	−	−	−	−	1	−	−	−	−	−	−	−	−	−	−	−	−	−	−	−	−	−	1	−	1	0.007
*Lu*. *longipalpis*	5	2	105	25	14	6	15	5	43	21	781	111	8	2	60	33	1	1	3	2	488	130	9131	1446	12	3	199	26	−	−	−	−	10865	1813	12678	99.614
*Ny*. *whitmani*	−	−	−	−	−	−	−	−	−	−	−	1	−	−	−	−	−	−	−	−	1	−	−	1	−	−	−	−	−	−	−	−	1	2	3	0.023
*Pa*. *bigeniculata*	−	−	1	−	−	−	−	−	−	−	−	1	−	−	−	−	−	−	−	−	−	−	−	1	−	−	−	−	−	−	−	−	1	2	3	0.023
*Pa*. *hermanlenti*	−	−	−	−	−	−	−	−	−	−	−	−	−	−	−	1	−	−	−	−	−	−	−	−	−	−	−	−	−	−	−	−	−	1	1	0.007
*Pa*. *punctigeniculata*	1	−	−	−	−	−	−	−	−	−	−	−	−	−	−	−	−	−	−	−	−	−	−	−	−	−	−	−	−	−	−	−	1	−	1	0.007
**Total**	6	2	107	25	22	6	22	7	43	21	782	114	8	5	62	37	2	4	5	3	490	132	9131	1450	12	3	199	26	−	1	−	−	10891	1836	12727	100

*Br: Brumptomyia; Ev: Evandromyia; Lu: Lutzomyia; Pa: Psathyromyia;* IN: indoors; OU: outdoors.

Outdoor collections had the highest number of sand flies comprising 94.1% (n = 11,970) of all sand flies collected. Only 756 sand flies, including 163 females were collected inside houses, and 95.4% were *Lu*. *longipalpis* (Table 1).

### Dominance

**Fig 1:**
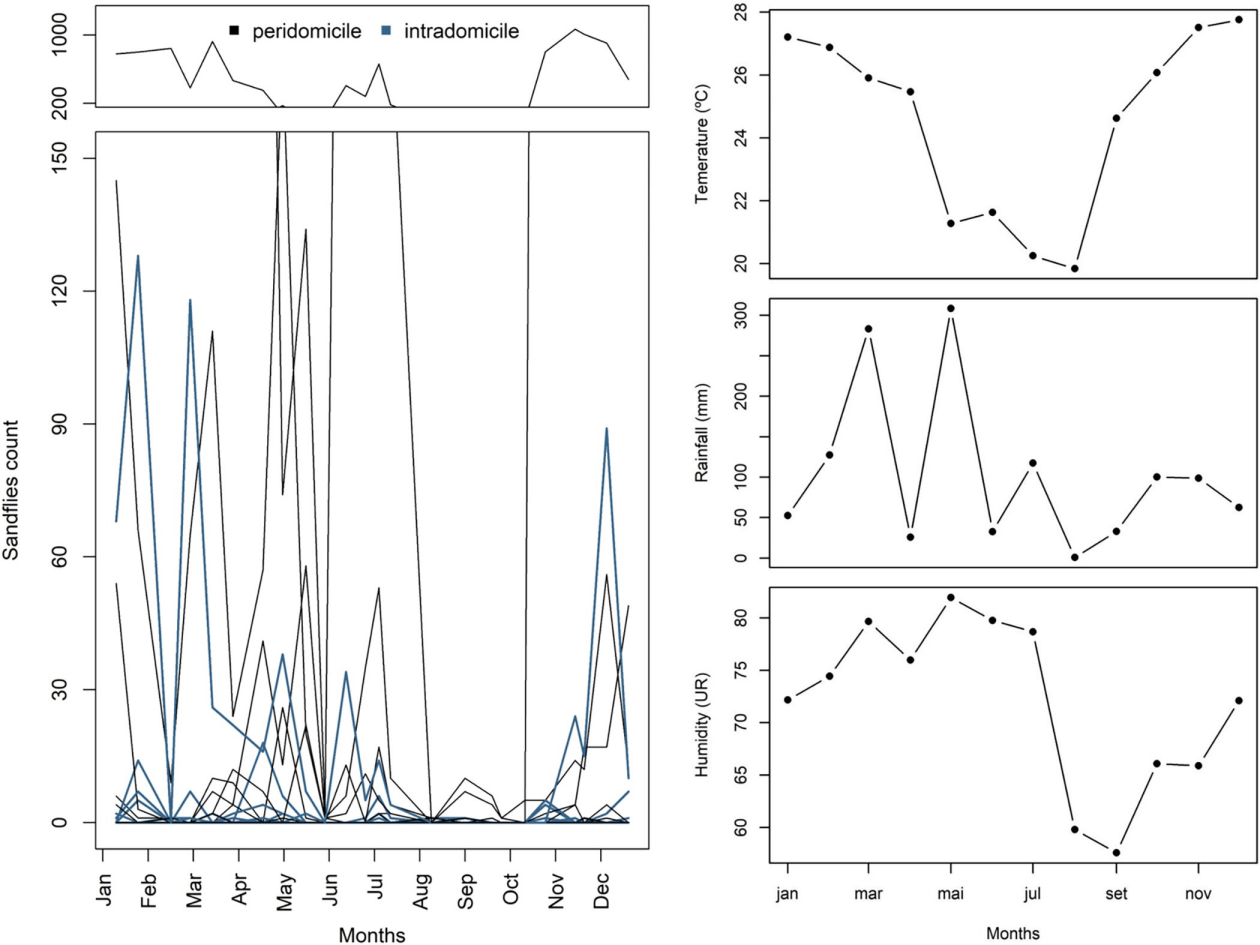
A (left panel): Average of sand fly abundance throughout the year by site and sampled location (outdoor[black] vs. indoor [blue]). B (right panel): Monthly mean temperature, humidity and accumulated rainfall throughout the year.

*Lutzomyia longipalpis* was present in both indoors and outdoors in seven out of nine sampled residences. The residence in site 6 (Nova Miranda neighborhood) was the site with the highest abundance of *Lu*. *longipalpis* accounting for 88.30% of *Lu*. *longipalpis* collected (Table 1 and Fig 1A).

*Lutzomyia longipalpis* was present all year round with greater abundance between November and March, the months with higher temperature and rainfall; 75.81% was captured indoors (Fig 1A).

**Fig 2:**
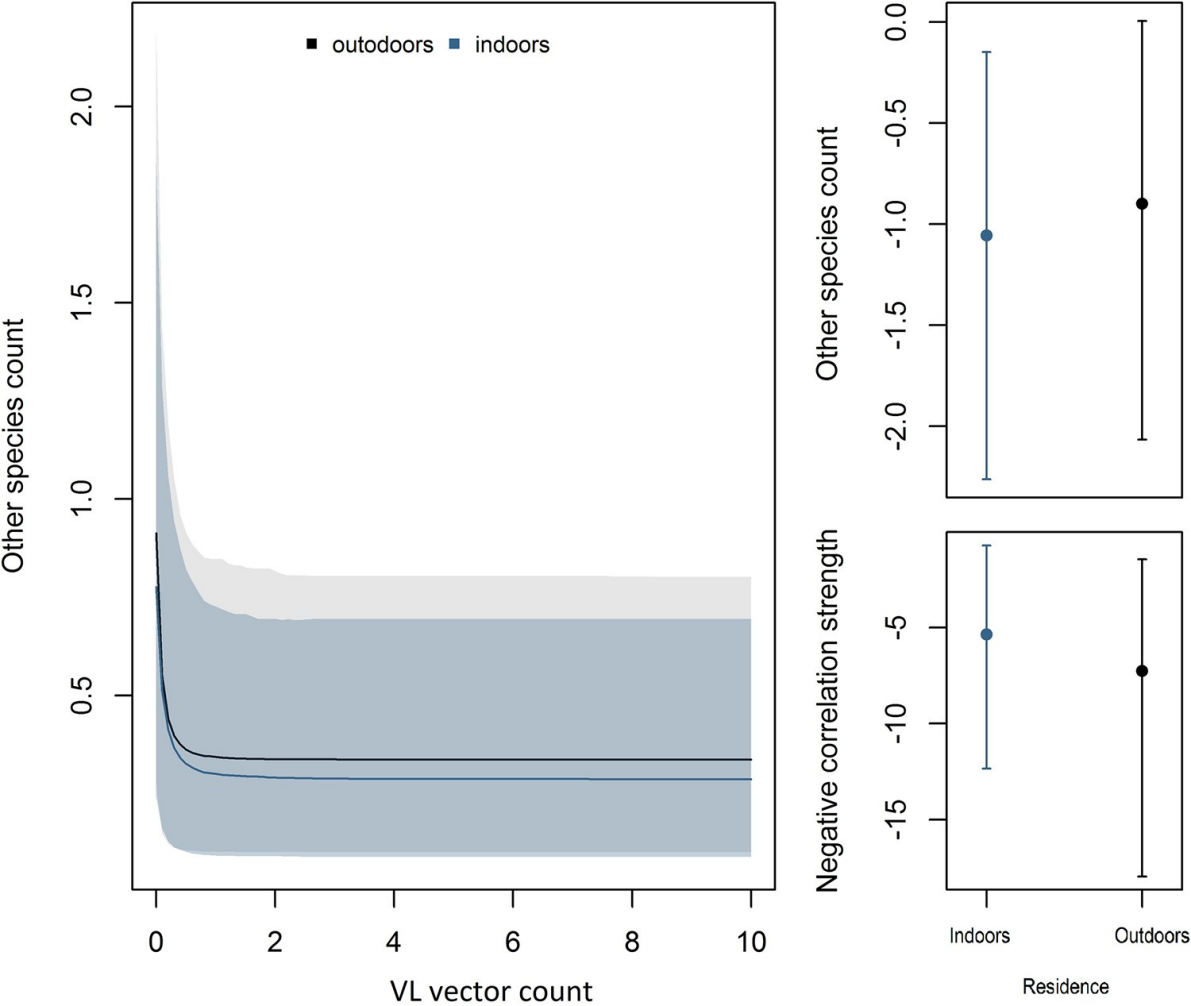
Exponential negative correlation between VL vector abundance and other sandfly species’ abundance (OSF) by sampling location (indoor/outdoors) (left panel). Other species count (model intercept) and negative correlation estimated for each domicile space (right panel).

We found OSF species abundance was very low, and presented a negative quadratic form, with abundance dropping to near zero from June to December (β_month_ = -2.26, IC95% = –21.86 to 15.90; β_month^2_ = -10.90, IC95% = –21.86 to 15.90), irrespective the residence space (β_month-outdoors_ = -0.58, IC95% = -19.71 to 18.37; β_month^2-outdoors_ = -3.97, IC95% = –21.10 to 11.06). Furthermore, OSF was negatively correlated with VL vector abundance, decreasing exponentially (β_VL_ = -9.92, IC95% = -25.05 to -0.06) as we observed the presence of the VL vector (Fig 2).

### Indoor versus outdoor exposure risk to VL vector through the year

**Fig 3:**
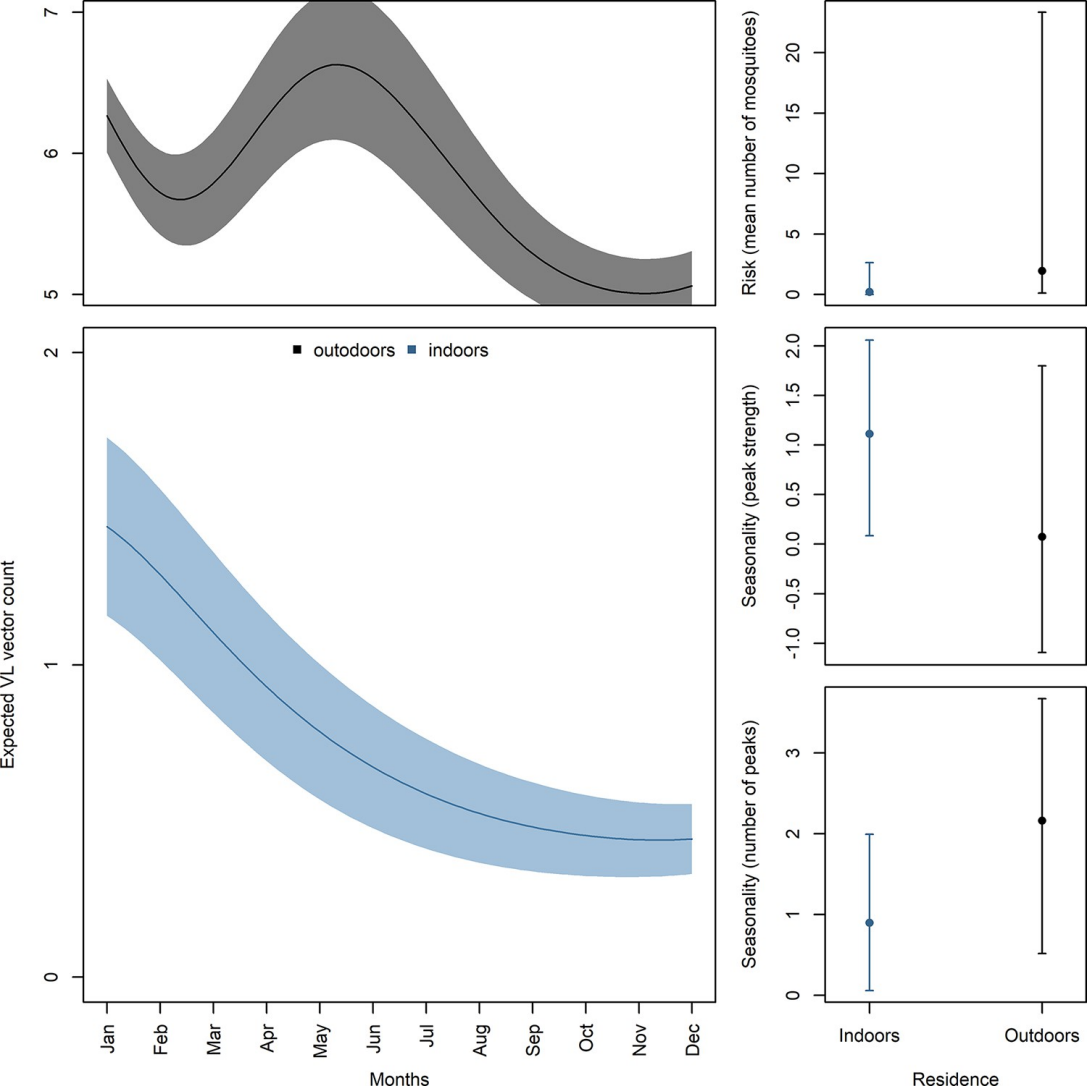
Expected seasonal changes in VL vector abundance throughout the year for each residence space (left panel). Risk, seasonality strength, and number of peaks estimated for each residence space (right panel).

VL vector abundance differed across residence space in terms of overall abundance and temporal variation throughout the year (Fig 3). Outdoor abundance was more than twice that observed indoors (*K*_outdoors_ = 2.23, IC95% = 1.31 to 2.90), and showed two distinct abundance peaks (January and June; *R*_outdoors_ = 2.13, IC95% = 0.13 to 4.24). In contrast, indoors only had one abundance peak (January; *R*_indoors_ = 0.90, IC95% = 0.06 to 1.99). Seasonality strength (relative size of a season peak) was similar between the sampled residence spaces (*E*_indoors_ = 1.10, IC95% = 0.08 to 2.06; *E*_outdoors_ = 0.22, IC95% = -2.18 to 3.10).

**Fig 4:**
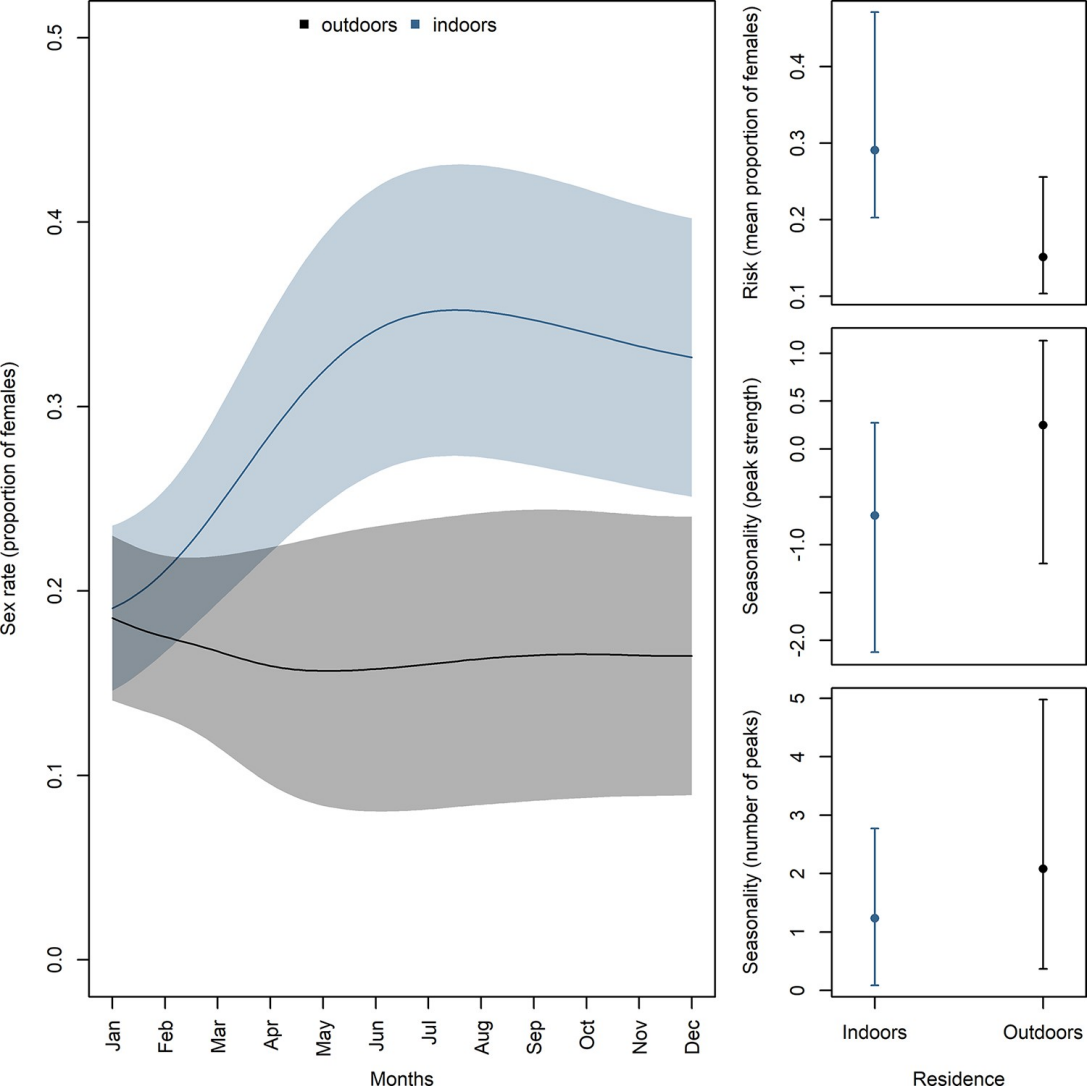
Sex ratio changes throughout the year by location (indoors vs outdoors) (left panel). Risk, seasonality strength, and number of peaks estimated for each location (indoors vs outdoors) (right panel).

As found for VL vector abundance, the proportion of females also differed inside and outside homes over the the year (Fig 4). However, the proportion of females found outdoors was consistently lower than found in indoors (*K*_outdoors_ = -0.84, IC95% = -1.35 to -0.55). Yet, estimated seasonality strength was equivalent but in opposite directions regarding the residence space: indoors was negative (*E*_indoors_ = -0.76, IC95% = -2.12 to 0.27), while outdoors positive (*E*_outdoors_ = 0.92, IC95% = 0.46 to 1.31). Finally, the indoors presented only one peak (June through August; *R*_indoors_ = 1.27, IC95% = 0.09 to 2.77), while outdoors presented two peaks (January-February, and August through October; *R*_outdoors_ = 2.34, IC95% = 0.10 to 6.31). Note, the combination of the different estimated parameters for each residence space revealed a very different profile of the risk of finding a female. Notoriously, the proportion of females outdoors presented a small decline after April, which was followed by an increase in the indoors’ proportion of females after May (Fig 4).

### Detection of *Leishmania* spp

From 1,240 samples of females analyzed by PCR, five tested positive for *Leishmania* spp. DNA. The minimum infection rate was 0.40%. *Leishmania* (*L*.) *infantum* was identified in pools with *Lu*. *longipalpis* from three different outdoors residences (01 Cherogamim, 02 Baiazinho, and 02 Nova Miranda) (Table 2).

**Table 2:** Females of sandflies submitted to PCR-RFLP for *Leishmania* DNA detection, number of positive pools, and minimum infection rate. Pools were organized per site, capture, and species, Miranda, Mato Grosso do Sul, Brazil, from August 2013 to July 2014.

Species	Number of females	Number of *Pools*	Positive Pools	MIR
*Ev*. *aldafalcaoae*	3	2	0	0
*Ev*. *evandroi*	6	4	0	0
*Ev*. *lenti*	5	3	0	0
*Ev*. *sallesi*	1	1	0	0
*Lu*. *longipalpis*	1,223	170	5	0.40
*Ny*. *whitmani*	2	2	0	0
TOTAL	1,240	182	5	0.40

*Ev*.: *Evandromyia; Lu*.: *Lutzomyia; Ny*.: *Nyssomyia;* MIR: Minimum infection rate.

## Discussion

Since 1912, Lutz & Neiva [26] have written about the adaptability of sandflies to environments associated with human habitations. Understanding their role in leishmaniasis epidemiology is crucial [27,28].

Sandflies fauna studies have been conducted in several regions of MS. In Miranda, Almeida et al. [29] captured *Ev*. *corumbaensis*, *Ev*. *sallesi*, *Ny*. *whitmani* and *Lu*. *longipalpis*, the last one the most abundant of them. In the same study *Br*. *avellari*, *Ev*. *aldafalcaoae*, *Ev*. *lenti*, *Ev*. *evandroi*, *Ev*. *walkeri*, *Pa*. *hermanlenti*, *Pa*. *bigeniculata*, and *Pa*. *punctigeniculata* were identified for the first time in this city.

The sandfly fauna in Miranda is very similar to Aquidauana and Corumbá, which are cities nearby. Species in common with Aquidauana are *Br*. *avellari*, *Ev*. *aldafalcaoae*, *Ev*. *evandroi*, *Ev*. *lenti*, *Ev*. *sallesi*, *Ev*. *walkeri*, *Ny whitmani*, *Lu*. *longipalpis* and *Pa*. *bigeniculata* [30,31] and with Corumbá: *Ev*. *sallesi*, *Ev*. *aldafalcaoae*, *Ev*. *walkeri* and *Pa*. *bigeniculata* [28,32]. Although *Lu*. *cruzi* was not captured in Miranda, it was the most abundant species reported in Corumbá. The connection of these three cities through BR 262 is one of the hypotheses of VL expansion from Corumbá to São Paulo [30,33].

In Miranda, *Lu*. *longipalpis* is present throughout the year and is clearly the most dominant species account of 99.6% of captured species. Other species only account for only 0.4% of the individuals trapped. Using the Generalized Negative Exponential Mixed Multilevel Model we observed a decrease in other species as the abundance of *Lu*. *longipalpis* increased. This negative correlation reinforced that *Lu*. *longipalpis* is totally adapted to an anthropogenic environment and could dominate over other species. This superior performance can be related to its eclectic food behavior and the ability to move and survive in urban areas [5,34]. The high prevalence of this sand fly in environments where the VL parasite is present highlights its importance in leishmaniasis transmission cycle [35]. It is noteworthy that *Lu*. *longipalpis* is more abundant in different environments, from rural areas like El Callejon, Colombia [36] to completely urbanized sites [6]. The abundance of this species is likely affected by the micro-habitat, in addition to the macro-environment characteristics [37].

The geographical distribution of *Lu*. *longipalpis* has a wide range and is still growing. Abundance of this species throughout Brazil has been recorded in VL transmission areas [18; 35]. Since the 1980s, its adaptation to the urban environment has been observed in Brazil. According to Salomon et al. [6], urbanization and dispersion involve a complex dynamic that includes climatic, environmental, and sociocultural aspects. The increase in insect dispersion and VL incidence in Mato Grosso do Sul has been attributed to environmental changes caused mainly by agribusiness [38,39].

This species is commonly captured with the highest number of males [27,40]. There are several hypotheses trying to explain, such as (1) the lekking behavior of which could attract first more males by kairomones followed by females; (2) As usually the traps are placed in animal shelters, females would be feeding while males would be more available and (3) the proximity between traps and breeding sites could attract more males once that emerge first of females [6,40,41].

Using a mixed multilevel model, we have demonstrated the risk of encountering the VL vector in both indoor and outdoor environments. The existence of domestic animals in the outdoor surroundings may be the cause of the high concentration of *Lu*. *longipalpis* in that area, as compared to the areas inside the forests, where these animals were mostly absent. In the Nova Miranda and Baiazinho neighborhoods, the sand fly prevalence was 95.56%, supporting the notion of their proximity to the dwellings. The same behavior has been observed in other regions where VL is endemic [30,33,42,43]. Salomon et al. [6] emphasizes that the area surrounding a house has important characteristics at both the micro and macro habitat scales for sandfly occurrence, including tree cover size, tree quantity, and organic matter accumulation on the ground.

The chicken coop had the most specimens and *Lutzomyia longipalpis* with 94.05% and 93.83%, respectively. Due to the significant number of insects found in Nova Miranda neighborhood, this site may be an important breeding location due to the availability of organic matter, primarily from chicken droppings. This high abundance of sand flies may be linked to *Lu*. *longipalpis*’ preference for feeding on chickens [44]. Although birds do not host the parasite, their presence can cause an increase in the breeding of phlebotomine near residences [33,45]. Chicken coop experiments by Forattini et al. [46] and Quinnel and Dye [47] have proven that *Lu*. *longipalpis* tends to be permanent and abundant in this type of environment.

On the other hand, there were many *Lu*. *longipalpis* found in inside residences (736 individuals), indicating the possibility of this species refuge inside homes increasing the possibility of them feeding on humans. As stated by Quinnel and Dye [48], proximity to animal shelters may facilitate sand fly entry into houses. In vulnerable areas, *Lutzomyia longipalpis* infestation tends to be higher, which increases the persistence and transmission risk of visceral leishmaniasis [49,50]. In Miranda, it was observed that the house next to a chicken coop without a ceiling had the highest number of individuals (618).

Besides using domestic animals as a food source, forested areas nearby may serve as resting sites and refuge for sand flies affect the frequency and abundance of sand flies in human environments [6,32]. This may be occurring in the Nova Miranda neighborhood, where forested areas bordering backyards could be maintaining the insects during chemical interventions for vector control measures performed by health authorities [5].

The abundance of *Lu*. *longipalpis* varied monthly, peaking from November to March when temperatures were at their highest. The year 2013 was anomalous, without drought. In the six months preceding the captures, 1,059 mm^3^ of rain was recorded. Among which, 422 mm^3^ and 230 mm^3^ fell in April and June respectively. Perhaps the insects’ behavior was impacted by the changes in rainfall, as the flooding in low Pantanal elevated the Miranda River level and the soil’s humidity. This scenario was similar in Campo Grande [5].

In this study, we captured infected females at sites with the highest numbers of *Lu*. *longipalpis*, namely Cherogamim, Baiazinho, and Nova Miranda. The sand flies’ minimum infection rate was 0.40%, which is relatively low compared to the range observed in other studies (from 0.0% to 3.9%) [5,24]. The detection of sand flies that are naturally infected with *Leishmania* spp. plays a crucial role in understanding leishmaniases epidemiology and vector competence [4].

As Miranda is a region where VL is endemic, discovering *Lu*. *longipalpis* females with DNA of *L*. (*L*.) *infantum* in the outdoors, particularly in areas with chickens, indicated that population dynamics of the vector are following natural infection. This finding implies that transmissions could be occurring within dwellings.

The presence of *Ev*. *sallesi* is also significant since it was found naturally infected with *L*. (*L*.) *infantum* [51]. Even though it is not anthropophilic, it could be part of the wild or rural cycle of leishmaniasis.

It is noteworthy to mention that *Ny*. *whitmani* was discovered in two chicken coops within the city. The species exhibits a remarkable ability to adapt to human-modified environments. Also, *Ny*. *whitmani* was confirmed as a carrier of *Leishmania* (*Viannia*) *braziliensis*, which is responsible for causing cutaneous leishmaniasis in Brazil [35,52,53].

Although we demonstrated the risk of residents contracting VL, we can improve our sampling and capture logistics. We plan to analyze the feeding habits of female sand flies and explore additional ecological aspects in future studies.

Miranda is a highly sought-after fishing destination in the Pantanal wetland located in the state of Mato Grosso do Sul. Due to the abundance of tourists from around the world who partake in fishing activities, insect bites are a concern. This is particularly alarming as Miranda is classified as a sporadic area for VL by SVS/MH [54] because it has experienced intense transmission as confirmed by our study.

## Conclusion

We found that although the sandfly fauna comprises 11 species, *Lutzomyia longipalpis* is the most prevalent species and is present year-round. The overall abundance of vectors outside was higher than inside the house. Despite a lower overall abundance indoors, the proportion of females was up to twice as high as that outdoors. Moreover, some females were found to be infected, indicating the circulation of *Leishmania infantum* in the area. These findings emphasize the spatial and temporal aspects of leishmaniasis transmission risk, which can aid in entomological surveillance and control measures. Therefore, the town should implement heightened health surveillance protocols, especially given its status as a popular fishing destination.

## Supporting information

S1 File Kml file with indicating the spatial distribution of sand fly sampling sites in the urban area of Miranda, Mato Grosso do Sul, Brazil. **Numbers 1–9 indicate sand fly sampling sites:** 01—Residence near farms, free-range chicken, reported human VL case in neighborhood.; 02—Residence near farms, domestic animals (pigsty, two chicken coops); 03—Residence within city, free range chickens, one dog; 04—Residence bordering forested area, free-range chickens; 05—Residence periphery of city chicken coop; 06 –Residence periphery, unkept chicken coop, bordered by wooded area; 07—Urban part of city, collected at residence of VL positive dog; 08 and 09—Sites furthest from urban center, near Miranda river, lots of native vegetation.
(KML)

## References

[1] Lainson R, Ward RD, Shaw JJ. Experimental transmission of Leishmania chagasi, causative agent of neotropical visceral leishmaniasis, by the sandfly Lutzomyia longipalpis. 1977; Nature. 266: 628–630. doi: 10.1038/266628a0 859627

[2] Sherlock IA. A Importância dos Flebotomíneos, In: Rangel E. e Lainson R. (Eds.), Flebotomíneos do Brasil. Rio de Janeiro, Fundação Oswaldo Cruz, 2021. pp 15–21.

[3] Lainson R, Rangel EF. Ecologia das leishmanioses Lutzomyia longipalpis e a eco-epidemiologia da leishmaniose visceral americana (LVA) no Brasil, In: Rangel E. F. e Lainson R. (Eds), Flebotomíneos do Brasil. Rio de Janeiro, Fundação Oswaldo Cruz, 2003. pp. 311–336.

[4] Oliveira EF, Oshiro ET, Fernandes WS, Ferreira AM, de Oliveira AG, Galati EA. Vector Competence of Lutzomyia cruzi Naturally Demonstrated for Leishmania infantum and Suspected for Leishmania amazonensis. 2017; Am. J. Trop. Med. Hyg, 11: 178–181. doi: 10.4269/ajtmh.16-0191 28077746 PMC5239689

[5] Oliveira AG, Galati EAB, Fernandes CE, Dorval MEC, Brazil RP. Seasonal variation of Lutzomyia longipalpis (Lutz & Neiva, 1912) (Diptera: Psychodidae: Phlebotominae) in endemic área of visceral leishmaniasis, Campo Grande, state of Mato Grosso do Sul, Brazil. 2008; Acta Trop. 105: 55–61. 18022137 10.1016/j.actatropica.2007.09.008

[6] Salomón O, Feliciangeli MD, Quintana MG, Afonso MMSA, Rangel EF. Lutzomyia longipalpis urbanisation and control. 2015; Mem Inst Oswaldo Cruz, 110(7): 831–846. doi: 10.1590/0074-02760150207 26517497 PMC4660613

[7] Andrade-Filho JD, Scholte RC; Amaral ALG, Shimabukuro PHF, Carvalho IOS, Caldeira RL. Occurrence and Probability Maps of Lutzomyia longipalpis and Lutzomyia cruzi (Diptera: Psychodidae: Phlebotominae) in Brazil. 2017; J. Med. Entomol. 54(5): 1430–1434. doi: 10.1093/jme/tjx094 28472338

[8] Barrios SPG, Pereira LE, Casaril AE, Infran JOM, Fernandes WS, Oshiro ET et. al. Phlebotominae (Diptera: Psychodidae) and biomes in the state of Mato Grosso Do Sul, Brazil. 2020; J. Med. Entomol. 57,: 1882 doi: 10.1093/jme/tjaa127 32804237

[9] Salomon OD. 2021. Lutzomyia longipalpis, gone with the wind and other variables. 2021; Neotropical Entomology. 50: p161–171. doi: 10.1007/s13744-020-00811-9 32840741

[10] Mota TF, de Sousa OMF, Silva YdJ, Borja LS, Leite BMM, Solcà MdS, et al. Natural infection by Leishmania infantum in the Lutzomyia longipalpis population of an endemic coastal area to visceral leishmaniasis in Brazil is not associated with bioclimatic factors. 2019; PLoS Negl Trop Dis 13(8): e0007626. doi: 10.1371/journal.pntd.0007626 31449534 PMC6730935

[11] Mato Grosso do Sul. Secretaria do Estado de Saúde, 2018. Informe Epidemiológico No 1/2018 Leishmaniose Visceral Mato Grosso do Sul.

[12] Solos Pott A., In: Pastagens no Pantanal. Corumbá, Empresa Brasileira de Pesquisa Agropecuária: Centro de Pesquisa Agropecuária do Pantanal, 1998 p. 18.

[13] Cadavid Garcia EA. Estudo técnico-econômico da pecuária bovina de corte do Pantanal Mato-Grossense. Corumbá, Empresa Brasileira de Pesquisa Agropecuária: Centro de Pesquisa Agropecuária do Pantanal, 1986: pp. 126–127.

[14] Campelo JH, Sandanielo A, Caneppele C, Priante Filho N. Climatologia. Diagnóstico dos meios físico e biótico: meio físico. Plano de Conservação da Bacia do Alto Paraguai (Pantanal)–PCBAP, In: Brasil. Ministério do Meio Ambiente, dos Recursos Hídricos e da Amazônia Legal. Brasília. PNMA, 1997: pp. 297–334.

[15] Falcão AR. 1981. Um novo modelo de armadilha luminosa de sucção para pequenos insetos. Mem Inst. Oswaldo Cruz 76: 303–305.

[16] Forattini O. P. 1973. Entomologia médica. Psychodidae, Phlebotominae, Leishmanioses, Bartonelose Vol. IV. Editora da Universidade de São Paulo, SP, Brazil.

[17] Galati EAB. 2023. Phlebotominae (Diptera, Psychodidae): classificação, morfologia, terminologia e identificação de adultos. Vol. I. São Paulo: Universidade de São Paulo; 2023. Disponível em: http://www.fsp.usp.br/egalati.

[18] Marcondes CB. A Proposal of Generic and Subgeneric Abreviations for Phlebotomine Sandflies (Diptera: Psychodidae: Phlebotominae) of the World. 2007; Entomol. News. 118: 351–356.

[19] Centro De Monitoramento De Tempo, Do Clima E Dos Recursos Hídricos De Mato Grosso Do Sul (Cemtec). [Cited 2014 Sept] Available from: http://www.agraer.ms.gov.br/cemtec/.

[20] Loxdale H, Lushai G. Molecular markers in entomology (Review). 1998; Bull. Entomol. Res. 88, 577–600.

[21] El Tai N, Osman F, El Far M, Presbefi W, Schönian G. Genetic heterogeneity of ribosomal internal transcribed spacer in clinical samples of Leishmania donovani spotted on filter paper as revealed by single-strand conformation polymorphisms and sequencing. 2000; Trans. R. Soc. Trop. Med. Hyg., 94: 575–79. doi: 10.1016/s0035-9203(00)90093-2 11132393

[22] Oliveira EF, Casaril AE, Mateus NLF, Murat PG, Fernandes WS, OSHIRO ET. et al. Leishmania amazonensis DNA in wild females of Lutzomyia cruzi (Diptera: Psychodidae) in the state of Mato Grosso do Sul, Brazil. 2015; Mem. Inst.Oswaldo Cruz, 110: 1051–1057. doi: 10.1590/0074-02760150317 26602870 PMC4708026

[23] Schönian G, Nascreddin A, Dinse N, Schweynoch C, Schallig HD, Presber W, Jaffe CL. PCR diagnosis and characterization of Leishmania in local and imported clinical samples. 2003; Diagn Microbiol Infect Dis. 47: 349–358. doi: 10.1016/s0732-8893(03)00093-2 12967749

[24] Paiva BR, Secundino NEC, Nascimento JC, Pimenta PFP, Galati EAB, Andrade Junior HE, Malafronte RS. Detection and identification of Leishmania species in field-captured phlebotomine sandflies based on mini-exon gene PCR. 2006; Acta Trop., 99: 252–259. doi: 10.1016/j.actatropica.2006.08.009 17055444

[25] Keane C, Marchetto KM, Oliveira-Santos LGR, Wünschmann A, Wolf TM. Epidemiological Investigation of Meningeal Worm-Indforatuced Mortalities in Small Ruminants and Camelids Over a 19 Year Period. 2022; Front Vet Sci. doi: 10.3389/fvets.2022.859028 35464381 PMC9020814

[26] Lutz A, Neiva A. Contribuição para o Conhecimento das Espécies do Gênero Phlebotomus Existentes no Brasil. 1912; Mem. Inst. Oswaldo Cruz. 04: 84–95.

[27] Casaril AE, Monaco NZN, Oliveira EF de, Eguchi GU, Filho ACP, Pereira LE et al. Spatiotemporal analyses of sandfly fauna (Diptera: Psychodidae) in an endemic area of visceral leishmaniasis at Pantanal, Central South America. 2014; Parasit. Vectors. 7:01–12. 10.1186/1756-3305-7-364 PMC4261527 25128480

[28] Fernandes WS, Borges LM, Casaril AE, de Oliveira EF, Infran JOM, Piranda EM et al. Sandfly fauna (Diptera: Psychodidae) in an urban area, Central-West of Brazil. 2017; Rev. Inst. Med. Trop. De São Paulo, 59: 1–8. 10.1590/S1678-9946201759054 PMC5574625 28902295

[29] Almeida PS, Nascimento JC do, Ferreira AM, Faccendo O, Filho JDA. Espécies de Flebotomíneos (Diptera: Psychodidae) Coletadas em Ambiente Urbano em Municípios com Transmissão de Leishmaniose Visceral do Estado de Mato Grosso do Sul, Brasil. 2010; Rev. Bras. Entomol. 2: 304–310.

[30] Figueiredo HR, Santos MFC, Casaril AE, Infran JOM, Ribeiro LM, Fernandes CE dos S et al. Sand Flies (Diptera: Psychodidae) in an endemic area of Leishmaniasis in Aquidauana Municipality, Pantanal of Mato Grosso do Sul, Brazil. 2016; Rev. Inst. Med. Trop. São Paulo, 58: 01–12. 10.1590/S1678-9946201658087 PMC5147717 27982353

[31] Galati EAB, Nunes VLB, Junior F de AR, Oshiro ET, Chang MR. Estudo de flebotomíneos (Diptera: Psychodidae) em foco de leishmaniose visceral Estado de Mato Grosso do Sul, Brasil. 1997; Rev. Saúde Públ. 31: 378–390. 10.1590/s0034-89101997000400007 9595767

[32] Antonialli SAC, Torres TG, Filho ACP, Tolezano JE. Spatial analysis of American Visceral Leishmaniasis in Mato Grosso do Sul State, Brazil.2007; J. Infect. 54:509–514. 16979241 10.1016/j.jinf.2006.08.004

[33] Oliveira AG, Galati EAB, Fernandes CE, Dorval MEC, Brazil RP. 2012. Ecological Aspects of Phlebotomones (Diptera: Psychodidae) in Endemic Area of Visceral Leishmaniasis, Campo Grande, State of Mato Grosso do Sul, Brazil. 2012; J. Med. Entomol. 49: 43–50. 22308770 10.1603/me11082

[34] Lainson R; Rangel EF. Lutzomyia longipalpis and eco-epidemiology of American visceral leishmaniasis, with particular reference to Brazil–A Review. 2005. Mem. Inst. Oswaldo Cruz. 100: 811–827. 16444411 10.1590/s0074-02762005000800001

[35] Infran JOM, Souza DA, Fernandes WS, Casaril AE, Eguchi GU, Oshiro E et al. Nycthemeral Rhythm of Phlebotominae (Diptera: Psychodidae) in a Craggy Region, Transitioning Between the Wetland and the Plateau, Brazil. 2016; J. of Med. Ent., 54: 114–124. 10.1093/jme/tjw151 28082638

[36] Ferro C, Morrison AC, Torres M, Pardo R, Wilson ML, Tesh RB. 1995. Species Composition and Relative Abundance of Sand Flies of the Genus Lutzomyia (Diptera: Psychodidae) at an Endemic Focus of Visceral Leishmaniasis in Colombia. J. Med. Entomol. 32(4): 527–537. doi: 10.1093/jmedent/32.4.527 7650716

[37] Tonelli GB, Binder C, Nogueira VLC, Prado MH, Theobaldo GG, Campos AM, et al. 2021. The sand fly (Diptera: Psychodidae) fauna of the urban area of Lassance, Northeast Minas Gerais, Brazil. PLoS ONE 16(10): e0257043. doi: 10.1371/journal.pone.0257043 34644289 PMC8513856

[38] Andrade ARO de, Nunes VLB, Galati EAB, Arruda CCP de, Santos MF da C et al. Epidemiological study on leishmaniasis in an area of environmental tourism and ecotourism, State of Mato Grosso do Sul, 2006–2007. 2009; Rev. Soc. Bras. Med. Trop. 42: 488–493. doi: 10.1590/s0037-86822009000500003 19967228

[39] Oliveira GMG, Filho EAF, Andrade GM de C, Araújo LA de, Oliveira MLG de, Cunha RV da. Flebotomíneos (Diptera: Psychodidae: Phlebotominae) no Município de Três Lagoas, área de transmissão intensa de leishmaniose visceral, Estado de Mato Grosso do Sul, Brasil. 2010; Rev. Pan-Amaz. Saúde; 1; 83–94.

[40] Ximenes M de FF de M, Souza M de F de, Castellón EG. Density of Sand Flies (Diptera: Psychodidae) in Domestic and Wild Animal Shelters in Area of Visceral Leishmaniasis in the State of Rio Grande do Norte, Brazil. 1999; Mem. Inst. Oswaldo Cruz. 94: 427–432. 10445997 10.1590/s0074-02761999000400001

[41] Jones TM, Hamilton JGC. A role for pheromones in mate choice in a lekking sadfly. 1998; Anim Behav. 56: 891–898. 9790700 10.1006/anbe.1998.0857

[42] Alexander B, Carvalho RL, de, McCallum H, Pereira MH. Role of the Domestic Chicken (Gallus gallus) in the Epidemiology of Urban Visceral Leishmaniasis in Brazil. 2002; Emerg. Infect. Dis. 08, 1480–1485. doi: 10.3201/eid0812.010485 12498667 PMC2738513

[43] Holcman MM, Sampaio SM, Rangel O, Casanova C. Spatial and seasonal distribution of Lutzomyia longipalpis in Dracena, a city in the western region of the state of São Paulo, Brazil, that is endemic with visceral leishmaniasis. 2013; Rev Soc Bras Med Trop 46(6):704–712. 24474011 10.1590/0037-8682-0188-2013

[44] Casanova C, Andrighetti MTM, Sampaio SMP, Marcoris MLG, Colla-Jacques FE, Prado AP.Larval Breeding Sites of Lutzomyia longipalpis (Diptera: Psychodidae) in Visceral Leishmaniasis Endemic Urban Areas in Southeastern Brazil.2013; PLoS Negl Trop Dis 7(9): e2443. doi: 10.1371/journal.pntd.0002443 24069494 PMC3777886

[45] Deane LM, Deane MP. Observações Sobre Abrigos e Criadouros de Flebótomos no Noroeste do Estado do Ceará. 1957; Bras. Malariol. Doenças Trop. 09: 225–246.

[46] Forattini OP, Rabello EX., Galati EAB. Novos Encontros de Flebotomíneos no Estado de São Paulo, Brasil, com Especial Referência à Lutzomyia longipalpis. 1976; Rev. Saúde Públ. 10: 125–128. 945607

[47] Quinnell RJ, Dye C. Na experimental study of the peridomestic distribution of Lutzomyia longipalpis (Diptera: Psychodidae). 1994; Bull. Entomol. Res. 84: 379–382.

[48] Quinnell RJ, Dye C. Correlates of the peridomestic abundance of Lutzomyia longipalpis (Diptera: Psychodidae) in Amazonian Brazil.1994; Med. Vet. Entomol. 08: 219–224. doi: 10.1111/j.1365-2915.1994.tb00502.x 7949312

[49] Michalsky EM, Guedes KS, Silva FOL, França-Silva JC, Dias CLF, Barata RC, Dias E.S. Infecção natural de Lutzomyia (Lutzomyia) longipalpis (Diptera: Psychodidae) por Leishmania infantum chagasi em flebotomíneos capturados no município de Janaúba, Estado de Minas Gerais, Brasil. 2011; Rev. Soc. Bras. Med. Trop. 44; 158–162. 10.1590/s0037-86822011000100014 21340410

[50] Vianna EN, Morais MHF, Almeida A.S, Sabroza PC, Reis IA, Dias ES, Carneiro M. 2016. Abundance of Lutzomyia longipalpis in urban households as risk factor of transmission of visceral leishmaniasis. Mem Inst Oswaldo Cruz, Rio de Janeiro, Vol. 111(5): 302–310 doi: 10.1590/0074-02760150366 27223866 PMC4878299

[51] Saraiva L, Carvalho GML, Gontijo CMF, Quaresma PF, Lima ACVMR, Falcão AL et al. Natural Infection of Lutzomyia neivai and Lutzomyia sallesi (Diptera: Psychodidae) by Leishmania Infantum chagasi in Brazil. 2009; J. Med. Entomol. 46: 1159–1163. doi: 10.1603/033.046.0525 19769049

[52] Diniz MMC de SL, Ovallo FG, Gomes CM de C, Lavitschka C. de O., Galati EAB. Host-biting rate and susceptibility of some suspected vectors to Leishmania braziliensis. 2014; Parasit. Vectors. 07: 01–11. doi: 10.1186/1756-3305-7-139 24684943 PMC3976554

[53] Pessoa SB, Coutinho JO. Infecção natural experimental dos flebótomos pela Leishmania braziliensis no estado de São Paulo. 1941; Hospital. 20: 25–35.

[54] Machado G, Alvarez J, Bakka HC, Perez A, Donato LE, de Ferreira Lima Júnior FE, Alves RV et al. Revisiting area risk classification of visceral leishmaniasis in Brazil. BMC Infect Dis. 2019; 19(1): 2. doi: 10.1186/s12879-018-3564-0 ; PMCID: PMC6318941. 30606104 PMC6318941

